# The invisible labor and multidimensional impacts of negotiating childcare on farms

**DOI:** 10.1007/s10460-020-10162-1

**Published:** 2020-10-14

**Authors:** Andrea Rissing, Shoshanah Inwood, Emily Stengel

**Affiliations:** 1 School of Environment and Natural Resources, The Ohio State University, 1680 Madison Ave, Wooster, OH 44691 USA; 2 Independent Scholar, New York, USA

**Keywords:** Sustainable agriculture, Family, Gender, Reproduction, Farm policy

## Abstract

Social science inquiries of American agriculture have long recognized the inextricability of farm households and farm businesses. Efforts to train and support farmers, however, often privilege business realm indicators over social issues. Such framings implicitly position households as disconnected from farm stress or farm success. This article argues that systematically tracing the pathways between farm households and farm operations represents a potentially powerful inroad towards identifying effective support interventions. We argue childcare arrangements are an underrecognized challenge through which farm household dynamics directly influence agricultural production. We draw on interviews and focus group data with farmers in the Northeastern United States to understand how farmer–parents access and negotiate childcare. Farmer–parents value raising children on farms, but express reluctance to expect current or future labor from them. Years with young children thus represent an especially vulnerable phase during a farm’s trajectory. We identify and analyze social, economic, and cognitive pathways through which childcare impacts farm operations. Social pathways include relationship tensions and gendered on-farm divisions of labor; economic pathways include farm layout and structure; cognitive pathways include how farmers think about and plan for their operations. Explicitly acknowledging such issues can better equip farmer–parents to anticipate and plan for conflicting demands on their time.

## Introduction

Although project specifics vary, a consistent logic unites most approaches to new farmer training. Niewolny and Lillard (2010, p. 73) found that while some holistic programming encourages farmers to reflect on their farming goals or personal values, overall, programs largely focus on “production practices, marketing, financial planning and resource assistance, business planning and management, and land acquisition and transfer.” Such programs aim to equip farmers with the information to make savvy business and production decisions, intending that these skillsets will prepare new farmers for agricultural success. However, Calo (2018, p. 376) argues that this ubiquitous emphasis on production and financial issues amounts to a “modern neoliberal vision of agriculture.” The predominate focus of identifying and remedying individual farmers’ proficiency gaps obscures important structural issues that benefit certain types of farms and disadvantage others.

This kind of individualistic programming—and the agricultural approach it represents more broadly—can also obscure the importance of household and community contexts for farm success. This article is, in part, a call for more systematic inquiries into under-recognized ways that farm household dynamics affect the trajectories, priorities, and functioning of agricultural production. To illustrate the value of such inquiries, we identify and analyze multiple ways through which childcare can impact farm operations.

The impetus for this research is two-fold. First, as social scientists working in American agriculture, we recognize that the importance of farm household processes for farm operations’ activities, growth, and viability consistently appears in the literature (e.g., Barlett 1993; Bennett and Kohl 1982; Fink 1986; Salamon 1992). However, farmer programming tends to implicitly ignore this insight by maintaining the household as a black box, keeping family issues separate from the enterprise and failing to integrate household needs and dynamics into farm enterprise planning. Additionally, much of the foundational research on farm family dynamics was conducted 40 years ago. Contemporary cultural shifts mean today’s farm household members have different backgrounds, priorities, parenting styles, and expectations than during the surge in social science interest in American farm viability during the 1980s farm crisis.

Second, over the combined three decades that we have studied American agriculture, a recurring pattern emerged in our conversations with farm families. Before field days, after interviews, during conference interludes, we heard variations on the same stories. We talked with people who found themselves simultaneously caring for elderly parents, young children, and a nascent farm business. Some felt the pressure of juggling young children and farm demands was too much and elected to pause their operation. Others quit farming after a divorce. Some young farmers shared that they decided not to have children for fear that they could not raise both a child and a farm. The years during which a farm family includes young children represent a particularly vulnerable phase for farm operations. Affordable, quality childcare can be a lifeline during this phase.

Childcare appears in both national policy and employer work-family policy level conversations (Hipp et al. 2017). Yet we rarely conceptualize parents who farm as ‘working parents,’ and few investigations have asked how childcare intersects efforts to strengthen farms and agricultural economies. However, childcare is a particularly salient issue as rural support systems and traditional institutions “hollow out” (Carr and Kefalas 2009), leading to community and family support gaps that can move through farm households to indirectly stress the country’s agricultural systems. Braun (2019) has recently identified the interplay of “ordinary” and “extraordinary” stressors on farms as catalyzing farm families’ high levels of stress. Private and public programming alike often provide needed supports in the face of extraordinary stressors, such as research on climate resilient agriculture or suicide-prevention hotline numbers. We suggest that considering how these issues intersect with ordinary stressors such as childcare arrangements more closely reflects the realities of farming.

To demonstrate how empirical research into farm household dynamics produces insights that can improve farmers’ quality of life and business functioning, we qualitatively analyze interviews and focus groups with 43 farmers in the Northeastern United States. Like other working parents, farmers with children face competing demands on their time. Childcare is not a one-off decision, but rather an ongoing process whose demands shift as families age and farm businesses expand. On family farms, the impacts of accessing and negotiating childcare flow outward from the household to affect farm operations. In this article, we identify three areas in which childcare decisions influence farm operations: *social*, *economic*, and *cognitive*. We envision these as three types of pathways stretching between farm households and farm operations, channeling the impacts of childcare discussions, decisions, and arrangements onto agricultural production in different ways. *Social* pathways include farmers’ identities and relationships; *economic* pathways include farms’ organization, structure, growth, and enterprise diversity; and *cognitive* pathways include how farmers plan for farm goals and think about farm timeframes. By charting such pathways, we illustrate the fundamentally interconnected nature of farm households and farm operations and indicate underrecognized areas for farm support interventions.

## Background

### Farm households, farm children, and childcare

At least since Alexander Chayanov’s analyses of Russian family farms’ demographic cycles (1966), social scientists have recognized the inextricability of household dynamics from the fate of farm operations. During and following the 1980s farm crisis, social science investigations into family farms’ functioning included many systematic examinations of kin relationships and household activities (e.g., Adams 1988; Barlett 1993; Bennett and Kohl 1982; Coleman and Elbert 1984; Reinhardt and Barlett 1989; Salamon 1992). Such studies argued that holistic accountings of farms require attending to the social contexts in which they are embedded. Today, American farm families from diverse geographic and production backgrounds consistently report lifestyle benefits, including flexibility, working outside and in tandem with nature, and alongside family members, as important motivators (Bruce 2019; Dreby et al. 2017).

Children have always been important components of farms’ social worlds. Although children historically worked on farms (Birk 2012; Effland 2005), increasing rates of mechanization in U.S. agriculture following the second world war (Barlett 1989; Fitzgerald 1991) reduced farms’ need for human labor. Even without contributing significant labor value, younger generations can embody the promised continuity of the farming operation. Transferring farm management and ownership is a fraught process that asks families to grapple with uncomfortable questions of death, competing visions, and equity (Goeller 2012). Nonetheless, many American farmers value keeping their farm in the family and hope to leave an operational farm to younger generations (Lobley and Baker 2012; Lobley et al. 2010; Valliant et al. 2017).

Farmer-parents are both part of and influenced by broader societal values and shifts, and contemporary farmers’ hopes and expectations for their children reflect current cultural discourses. After decades of urbanization, the unique benefits of raising children on working farms have become more desirable, and many new farmers express a desire to live and work on farms with their children (Johnson et al. 2001). Drawing on Zelizer’s (1985) analysis of how, during the twentieth century, American children moved from being “economically useful” to their family units towards being “emotionally priceless” to their parents, Dreby and Carr (2019) argue that producers within the “modern farming movement” choose to pursue agriculture largely because of the attractive environment it offers for raising children. Farming is a hard career in which to make a living, but many farmers feel the educational benefits of on-farm childhoods offer intangible yet vital life-long advantages for their children, regardless of whether they take over management one day.

Children gaining these valuable life skills and practical knowledge on farms still need to be cared for. This issue comes to a head in particularly tense ways when operators strive to run viable businesses. Who provides the care? Gendered divisions of labor have long characterized the work rhythms of American farms (Fink 1986; Rosenfeld^1987^; Sachs 1996), and childcare is especially prone to such patterns. Gendered work roles on farms mimic the greater labor force in the U.S. As more women joined the workforce in the 1970s and 1980s, changes in traditional domestic labor responsibilities were slow to follow (Clawson and Gerstel 2014; Hochschild and Machung 2003[1989]). Women still bear the “lion’s share” of household chores (Lachance-Grzela and Bouchard 2010), childcare (Bianchi et al. 2012), and family emotional work (di Leonardo 1987; Erickson 2005).

On farms, women also experience this stress as their time stretches between farm and household responsibilities (Berkowitz and Perkins 1984; Sachs et al. 2016). Researchers focusing on gender’s intersections with farm work have, (1) interrogated the competing identity labels of “woman farmer” and “farm wife,” (Brasier et al. 2014; Fink 1992; Sachs 1996; Whatmore 1991); (2) demonstrated that women farmers’ needs are often unmet by formal agricultural education and technical assistance (Barbercheck et al. 2009; Brasier et al. 2009; Trauger et al. 2008); and 3) shown how sustainable agriculture spaces have been more affirming of women farmers’ values and goals (DeLind and Ferguson 1999; Hassanein 1999; Trauger 2004).

Within this recent feminist scholarship, however, farm household reproduction has gone largely unexamined. Although not all farm children will grow up to be farmers, ensuring the care of farm households’ youngest members remains an activity with far-reaching implications for agriculture that falls largely on farm women. These dynamics are not unique to the United States or to farm households. Recently, organizations such as ActionAid and Oxfam International have called attention to the inequitable distribution of unpaid care work and its role in entrenching poverty in developing countries (Coffey et al. 2020; Coffey and Staszewka 2017). Feminist economists have for decades anticipated these calls by arguing that the un- or under-paid care work of social reproduction constitutes an invisible subsidy to the formal economy, often provided by women (Donath 2000; Himmelweit 1995; Power 2004). Childcare is a fundamental, yet undervalued, component of household economics. Translating this connection onto farms reveals childcare as an underrecognized factor within agricultural economics. Scholarly efforts to understand the lived experiences of agriculture and practical efforts to support farmers’ quality of life alike must take this element of farm work into account.

### Childcare for U.S. working families

Although care work often falls to the women in a household, many families also look to external childcare providers. Accessing affordable, high-quality care challenges families across socioeconomic backgrounds, geographic locations, and professions (Forry 2006; Morrissey 2008; Walker and Reschke 2004). However, families’ abilities to secure the kind of childcare they desire depends in part upon the policy landscape in which they live. Attempts to create a coherent national childcare program in the U.S. have included daycare for low-income mothers, mothers’ and widows’ pensions, and a childcare tax deduction (Michel 1999). Despite these attempts to address the childcare needs of working families, political divisions in the U.S. have prevented the passage of Western-European style public social support systems. Such systems provide comprehensive childcare and family policies, with generous parental leave and subsidized care available to all parents (Morgan 2006). In the U.S., the Comprehensive Child Development Act (CCDA), or Mondale-Brademas bill, of 1971 came closest to creating a universal, publicly supported childcare program. The CCDA, which would have granted low-income families access to free childcare and made these services available to other families on a sliding fee scale, passed both Houses of Congress, but was vetoed by President Nixon (Berry 1993; Dinner 2010; Michel 1999).

Today, federal support for childcare includes subsidies and tax deductions. The Childcare and Development Block Grant, created in 1990 as the Childcare Development Fund and reauthorized by President Obama in 2014, subsidizes the purchase of private-market childcare for low-income parents via a finite number of vouchers issued to families at the state level. As an alternative, the Child and Dependent Care Tax Credit, available to all families, covers a portion of employment-related childcare (Internal Revenue Service 2020). However, states establish their own childcare licensing regulations, such as mandating child-staff ratios. This leads to wide variance in parents’ experience with childcare from state to state (Davis and Connelly 2005; Davis and Li 2009).

Despite public funds for childcare, longstanding issues of supply and quality continue to affect the choices of all families seeking childcare. Childcare services fuel economic development in all sectors, but they have also been widely undervalued and feminized (Hoffer 2002; Warner 2009). This, combined with the tendency to discount the value of informal or unpaid (e.g., familial) childcare, has resulted in narrow conceptual and political approaches to childcare which can over-emphasize the role of formal licensed providers (Stoney et al. 2006; Warner 2009). Although its provision often does rely upon the private sector, childcare is also embedded in social structures and community networks, where parents receive most of their information about the location, supply, and quality of care options (Meyers and Jordan 2006).

More broadly, today’s economy blurs the traditional categories of “full time,” “part time,” and “nonstandard” work hours (Clawson and Gerstel 2014). In effect, more sectors of the economy reflect the intermingling of work and life, non-traditional hours, and income precarity that has long characterized agriculture. Examining the household economics and livelihood strategies underpinning farm operations provides a timely window through which to understand this social issue and its relationship to economic development and public policy (Lobao and Meyer 2001).

### Childcare for farm families

While childcare in agriculture presents a useful lens for examining larger social issues, as an industry, agriculture also faces unique challenges. One important area in which on-farm childhoods differ from non-farm ones is safety. Farms can be hazardous places for children of farmworkers and farm owner-operators (Centers for Disease Control and Prevention 2020). Caring for children on farms, particularly when industrial equipment is present, entails different kinds of supervision than suffices in non-farm settings (Morrongiello et al. 2012; Liebman et al. 2019), and parents must regularly weigh the risks and benefits of children’s on-farm presence (Elliot et al. 2018). Researchers have recently called for improved childcare access for farmworker parents to help prevent pediatric injuries (Liebman et al. 2014; Miller et al. 2016). Language and transportation issues compound the cost and safety challenges for seasonal and migrant farmworkers (Liebman et al. 2019), leading some to suggest agri-business leaders and employers consider supporting childcare as a workforce investment (Lee et al. 2017; Liebman et al. 2017). While there have been notable efforts to research and practically address childcare needs for farmworkers, there has been substantially less investigation into the childcare needs of farm owner-operators, or how childcare affects farm development.

Farm families can also struggle to find affordable, high quality care. The current system of childcare subsidies and employment-based tax credits may not always match farm families’ needs, especially those with nontraditional hours, off-farm jobs, or who prefer informal care or family caregivers (who may or may not be licensed providers). Reschke (2012) notes that childcare provided by neighbors or family can more easily accommodate nontraditional hours than formal daycare centers and is particularly relevant for farm families and others whose work schedules may require flexibility. Parents who work nontraditional hours are more likely both to utilize informal care and to use multiple childcare providers (Presser 2003).

Geography can also complicate farm families’ access to childcare. Driving to a daycare center may take longer for rural families in more isolated locales or smaller towns. Discrepancies in childcare availability, affordability, and government support also exist between rural and urban areas. Reschke (2012) suggests such discrepancies may be attributed to lack of access to formal care, as well as to nontraditional work. Studies have consistently found that family care is an attractive option for rural families (Reschke et al. 2006); however, farmers who start businesses in locations without relatives nearby may not easily access family care.

Finally, low and fluctuating returns may make it difficult for farm families to afford off-farm care, particularly for the small family farms that predominate national and Northeastern farm landscapes alike (USDA NASS 2019b). Small farms, those reporting annual gross income of less than $250,000, are much more likely to rely on off-farm income (Hoppe et al. 2010), which can both necessitate and complicate childcare.

## Approach and methods

This study was designed as an exploratory inquiry to understand the use of childcare by the farming population in the Northeast. The study addressed three research questions: (1) How does childcare affect farm businesses and farm families? (2) What childcare arrangements are farm families making? (3) What strategies do farm families identify as solutions to childcare challenges? This article’s analysis focuses on the first research question. Data were collected between 2014 and 2015. See Inwood and Stengel (2020) for a quantitative analysis of farmers’ childcare experiences drawn from a concurrently conducted national survey.

A purposive sample of farmers with children were invited via agricultural organizations’ listservs, newsletters, social media, and direct contact to participate in focus groups in nine Northeastern states: Connecticut, Maine, Massachusetts, New Hampshire, New Jersey, New York, Pennsylvania, Rhode Island, and Vermont. Snowball sampling through participants and professional contacts helped identify additional participants. Four focus groups were ultimately held in New Jersey, New York, Pennsylvania, and Vermont. Seventeen farmers participated in focus groups, and each focus group ranged in size from three to five participants. Focus groups lasted ninety minutes, during which time the third author led participants through a series of questions regarding their farm vision, children’s on-farm roles, goals for their children, forms of childcare used and desired, childcare’s effects on farm families and farm business, and available and desired childcare support systems.

Interested farmers unable to attend focus groups^1^ were invited to participate in either in-person or phone interviews. From this group, the third author conducted twenty-six additional individual interviews, covering the topics described above. Twenty of these interviews were held in person, and six were conducted over the phone. Interviews typically lasted between 30 and 60 min, although a few went as long as two hours. Both focus groups and interviews were semi-structured with pre-determined open-ended questions that allowed flexibility to digress or probe further and clarify based on the individual’s responses. All participants received a rain gauge.

We present findings from focus groups and interviews together. We used very similar instruments for both methods and, as the topics covered are only moderately sensitive, we can reasonably expect that respondents would answer similarly in either setting (Wutich et al. 2010). Focus group and interview transcripts were inductively double-coded following Braun and Clarke’s (2006) method for qualitative analysis to identify salient themes and patterns.

Table 1 summarizes participants’ demographic information. The sample includes thirty-three women and ten men. We did not explicitly ask about sexual orientation; all participants referenced opposite-sex partners. Forty-one participants identified as “White, non-Hispanic/Latino,” and two identified as “White, Hispanic/Latino.” Our participant group’s racial homogeneity stems from several interrelated reasons. While the Northeastern United States is home to a diverse population of Latinx farmworkers (Mares 2019), USDA census data indicates that the vast majority of principal farm operators in the Northeast are white. For example, according to the most recent census, white producers operated 6797 of Vermont’s 6808 farms (USDA NASS 2017). Although these statewide statistics likely reflect a systematic undercounting of Latinx farmers due to structural and linguistic barriers (Minkoff-Zern 2019), our study’s recruitment nonetheless occurred in primarily white spaces. In particular, the 2014 conferences at which focus groups occurred were mostly attended by white farmers, indicating that the earlier listserv and social media recruitment likely also inadvertently targeted primarily white farmers through these networks. In addition to convenience sampling, we also employed snowball sampling, and participants exclusively referred us to other white farmers. The lack of racial diversity in our sample evinces the effects of systemic racism within the U.S. agriculture’s sector’s lending, land inheritance, outreach and technical assistance, and other practices (Daniel 2015; Horst and Marion 2019; Quisumbing King, et al. 2018). Convenience and snowball sampling’s failure to produce a diverse participant group speaks to inherent limitations of these methods and to the ongoing effects of structural racial discrimination in agriculture. The homogeneity of this sample limits our findings’ generalizability to the U.S. farming population.

**Table 1:** Participants in the study

	Male		Females		Total	
	*n* = 10		*n* = 33		*N* = 43	
	Cases	%	Cases	%	Cases	%
Age						
21–30	1	10.0	3	9.1	4	9.3
31–40	6	60.0	21	63.6	27	62.8
41–50	2	20.0	7	21.2	9	20.9
51–60	1	10.0	2	6.1	3	7.0
No. of children						
0	1	10.0	3	9.1	4	9.3
1	5	50.0	11	33.3	16	37.2
2	3	30.0	13	39.4	16	37.2
3	1	10.0	4	9.1	5	11.6
4	0	0.0	2	6.1	2	4.7
Family history						
Multi-generation	0	0.0	9	27.3	9	20.9
first generation	10	100.0	24	72.7	34	79.1

Most participants have either one or two children. One participant has custody of grandchildren and one has had foster children. Four of the participants did not have children at the time of the study. Three were planning for children in the near future, and one cares for foster children but did not have custody of any during the study. These participants are included under ‘Number of Children’ as “0.” Over three-quarters (79.1%) of participants were *first-generation* farmers, lacking a family farm background, while less than a quarter (20.9%) of participants were *multi-generation* farmers who had grown up on a farm. First-generation and multi-generation are distinct categories from *beginning farmer*, which the USDA defines as someone who has operated their farm for less than 10 consecutive years.

Forty-three participants are included in the analysis. Three participants were in the process of both establishing their farms and planning for their first children; they provide perspectives as both future farmer and future parent. Three couples were interviewed, either together or separately. Accounting for these cases leaves 37 active farms with children. Using the USDA’s classification of farms by gross cash farm income (Hoppe et al. 2010), the majority of participants have small commercial farms (between $10,000 and $250,000 in annual farm sales), and seven have large farms (greater than $250,000 in annual farm sales). The Northeastern U.S. is dominated by dairy and mixed-crop operations, is densely populated with small farms, and has a higher concentration of female farmers than the national average (USDA NASS 2019b). This study’s participants primarily ran direct-market farms, more than half of which were diversified vegetable operations (Table 2).

**Table 2:** Farms in the study

	N = 37	
	Cases	%
Farm size		
Small commercial farm	30	81.1
Large farm	7	18.9
Farm type		
Vegetables	20	54.1
Vegetables and livestock	6	16.2
Livestock	3	8.1
Dairy	3	8.1
Other	5	13.5

## Findings

In the remainder of this article, we first examine the diverse childcare strategies employed by study participants as they seek to balance farm work and caring for children. We then present findings on participants’ future expectations for their children and analyze the tension between wanting to raise one’s children on a farm without assuming they will farm themselves someday. In the second half of this section, we describe three pathways through which childcare links farm households and farm operations. As farmers negotiate childcare access over time, the impacts of these decisions flow along *social*, *economic*, and *cognitive* pathways from the farm households towards the farm operation to affect it along multiple dimensions.

### Who watches the kids when you work?

Reflecting their diversity, participants drew upon a range of strategies to meet their families’ needs while striving to honor the values that had originally brought them to farming. Negotiating these competing household-level issues, in turn, affected both the daily operating and long-term visioning of the farming operation. We first asked participants, “What do your children do while you are farming, marketing, recordkeeping, or other farm-related tasks?” Responses were categorized into six groups inspired by the childcare typology used by Warner (2007): *off farm formal*, which includes formal daycare centers; *off farm informal*, including home care or care at a neighbor or friend’s house; *on farm informal*, such as a babysitter; *parental care*, which includes children at home or on the farm with their parents; *family care*, such as a grandparent; and *nontraditional care*, which includes nanny sharing and co-operative style childcare. Participants pieced together childcare through many forms. All reported using at least two different types of care.

All 37 families reporting using parental care as one childcare method. Despite its popularity among participants, parental care can disrupt productivity. One first-generation male participant, whose wife works off the farm, had hoped for his young son “To be my little sidekick and … do everything I did.” However, he found reality was much different. He admitted that he “Didn’t think about a baby not being able to be out in the sun all day,” and, at the time of the interview, struggled to balance care work and farm work.

Off-farm formal care was the second most popular method among participants, with 21 out of 37 families reporting using it alongside other methods. Seven participants have accessed childcare subsidies for this type of care, either through reimbursed care or state sponsored Head Start programs. Participants reported both increased on-farm productivity and the desire for children to socialize off-farm as factors influencing their choice. “We don’t want our kids to be isolated,” explained one first-generation female farmer.

Importantly, not all farmers who desired off-farm care could access it. Rural areas often suffer from a scarcity of convenient essential services, and rural residents often must travel further than urban residents to access healthcare, financial services, or retail (Brundisini et al., 2013; Thiede et al., 2017). Childcare access in rural areas seems to follow this pattern. As one participant explained, “You get a farm, especially if you’re young, where you can afford it. And where you can afford a farm is not going to be a place where there’s a lot of resources.” For her, affording off-farm childcare was less of an issue than the half-hour drive it required; more than the financial costs, she could not afford the time out of her day.

Family care is the third most frequently reported type of care, with 19 of 37 families reporting that they relied on family members to provide care. This includes care by grandparents or other relatives and can be on or off the farm. Family care was the most desired care arrangement among all participants. Multigenerational farmers and participants with large farms reported using this type more than any other, noting they often chose it for both financial reasons and the quality of care. Family care’s flexibility also proved valuable given agriculture’s nontraditional work hours.

On- and off- farm informal care and non-traditional care were less common, reported by 10, 6, and 8 respondents, respectively. The childcare arrangements made by participants reflect tensions between expectations and reality, and are influenced by families’ values, farm productivity, cost of care, and distance to care centers and relatives.

### Farmer–parent motivations and expectations: “I’m not raising a small farmer”

To understand the stakes of childcare access on farms, we begin where farm parents do: with the intense desire to raise their children on farms. Many studies have documented farmers’ motivations to keep the farm within their family and pass on to their children the land and business they inherited (Barlett 1993; Inwood et al. 2013; Salamon 1992). Perhaps reflecting this sample’s high percentage of first-generation farmers, the farmers interviewed for this study were more reticent to express a clear vision that their children would farm themselves one day. These findings echo those of Dreby and Carr (2019, p. 14), who argued that farm parents kept “expectations of future involvement low” as part of their emphasis on the farm’s ability to serve children’s education needs, as opposed to children serving the farm’s economic ones. In our study, parents generally expressed hopes that their children would find exciting and meaningful careers, but they were uninvested in whether this future vocation was agricultural.

In a review of parenting styles in the United States, psychologists Tamis-Lemoda and McFadden (2010, p. 300) describe “the ubiquitous characterization of U.S. parenting and child development as ‘individualistic.’” Although variation certainly occurs, this individualism can manifest as parental desire to support children in making their own choices, embracing autonomy, and reaching their full potential. Among this project’s participants, this approach to parenting seemed widely embraced. In response to a question about long-term goals for children, a farmer who produces artisan cheese with her husband replied, “Well, my long-term goals are just for her to find her passion and do what she’s passionate about, so our focus has been to let her be a well-rounded person and do what she wants to do. If she wants to farm, hopefully, it’ll be there.” In discussing their openness to a range of career paths for their children, other participants emphasized their children’s own decision-making, saying, “Maybe he’ll be a ballerina” or “They may decide they want to be stockbrokers.”

Although participants generally declined to express visions of their children as adult farmers, they firmly believed that on-farm childhoods provide myriad unique benefits. Indeed, several participants mentioned raising their children on a farm as one of their own motivations for farming. Participants frequently emphasized that, regardless of what the future held, the experience of growing up on a farm would instill greater knowledge of ecological processes, appreciation for meaningful work, and more profound connections to the natural world. Farmers expressed hopes that endowing their children with these embodied characteristics would yield diverse benefits.

Farmer–parents often expressed concern that forcing children’s participation in farm activities would lead to rebellion or resentment. One participant ran a certified organic vegetable operation that employed five people full time year-round, plus ten additional seasonal employees. Despite their clear labor need, she and her husband had no plans to rely on their school-aged children. She said, “We decided pretty early on that they wouldn’t be required to work. We wanted them to do it because they want to and want to participate. The farm is very consuming in our life and so I didn’t want them to feel like they were obligated to be in it all the time.” During a focus group, a man who had raised his now-adult children on the farm similarly explained, “You can’t force them to do it. If you force them to do it, they’re going to hate it and then you’re just going to have all kinds of headaches down the road.”

Multiple trends of modernity converge in these farmer–parents’ lives. When less than 2% of Americans are employed in agriculture, these parents feel they are giving their children something precious through the opportunity to, as one Vermont farmer put it, “Grow up outside and [understand] all about how foods grow.” Simultaneously, parents want to honor and support their children’s individual passions and empower them to choose their own path. Thus, although participants mentioned the intermingling of farm work and child rearing as desirable, in practice, these two areas often became competitively opposed. Echoing Hochschild’s (1997) “time debt,” where employed parents felt they owed their children after working long hours outside the home, farmer-parents often described their time as a finite resource to dole out between the farm and their children. Allocating personal resources towards one always came at the expense of the other.

Some participants spoke of having to compromise between attending to children’s needs and farm needs. One farmer lamented, “I’ve got seven employees and I’m the manager, for lack of a better word. And my child is starving, and I have to decide, do I put the farm first or my child first. And I’m always going to choose my child. But then I’ve got seven employees wandering around not knowing what to do next.” Another farmer described the tension as one of competing identities: “I love being with [my daughter], I love being a mom but I also love being a farmer. I feel like I’ve had to give some of that up, and I don’t want to lose that.”

This sample of farmer–parents expressed highly varied ways of conceptualizing and accessing childcare as a means to achieve their farm and family goals. Participants both deeply valued raising their children on farms and typically shied away from demanding or expecting their children have any specific engagement with the farm. The affective backdrop of childcare decisions comprises a tension between, on one hand, parents’ conviction that farming helps children gain rare life skills and, on the other hand, a reluctance to use one’s children as farm labor or expect them to share one’s own vocational calling. As one vegetable producer stated unambiguously about her young son, “I’m not raising a small farmer.” By allowing children to be raised on farms without necessarily being involved in all production areas at all times, childcare may help mediate this tension.

### Farm households impacting farm operations: *social*, *economic*, and *cognitive* pathways

Accessing and negotiating childcare also impacts the farm operation. In order to understand how childcare, an activity that seems primarily associated with households, can carry wide-ranging impacts for agricultural production, we identify three areas of interest: *social*, *economic*, and *cognitive*. In the remainder of this section, we present these areas as interrelated pathways that channel the impacts of childcare onto farm operations.

#### Social

##### Mom leaves the fields

Thanks to targeted supports for historically underrepresented groups, positive platforms such as The Female Farmer Project, and the Census of Agriculture now accommodating multiple principle operators, the number of recorded women farmers has risen in recent years. As of the 2017 Census of Agriculture, 9% of all U.S. farms were operated exclusively by women, and 56% of U.S. farms reported at least one female producer (USDA NASS 2019a). However, increasing a sector’s gender diversity does not guarantee equitable labor distribution. Like most American households, gendered divisions of labor continue to characterize many farm households. Farm women handle an outsize amount of household and care work. Our research suggests that the familial dynamics unfolding as childcare needs arise on farms can accelerate this process.

In this study, some mothers described how, during their children’s early years, they took on more caring work. This increase in parental care responsibilities sometimes necessitated decreasing their involvement in physical fieldwork, which could be difficult to undo. One participant told the story of her family’s farm beginning as a joint venture where she and her husband contributed equally to all facets of the business. She described how they had originally fallen in love while doing fieldwork together, but now, after having three children, she no longer finds herself in the fields. Echoing a common postpartum evolution of labor responsibilities, she reported, “Our roles in the farm have shifted dramatically.” Where her husband now manages the farm production, she manages the administrative side of the business. She used to find fulfillment from working with crops, but the time she spent away from physical labor while caring for small children has created a lasting barrier:

It’s very hard for me to jump into working with the crew… I’m not in the farming shape that I used to be… It became harder for me to feel like I could just jump in because I felt so far behind the crew. Because I wasn’t out in the field, I didn’t always know where things were.

Other female participants echoed similar changes in farm roles as a result of caring for children. Some reported resulting stress from trying to negotiate the divergence of historical expectations of women on the farm and the reality of women’s farm roles today. One female farmer, surprised by the gendered roles she and her husband assumed once they had children, explained:

There’s always been kind of the farm wife who cooks and cleans and takes care of the kids while the farmer goes out and works in the fields. That is shifting. There’s a lot more women farmers. But they’re still responsible for the childcare and the cooking and the cleaning and they don’t have a farm wife to take care of that. They’re a wife and farmer and it’s really challenging.

Though many participants explicitly discussed changes in gender roles and parental relationships, there was also a sense that these farmer–parents experienced childcare differently than they had originally hoped for. A female farmer reported a commonly expressed dissonance between her expectation of the division of labor and the reality:

There’s often a division of labor, sort of a stereotypical male/female division of labor, which is not at all how we intended for our lives to go…I envisioned that we would be able to work more with our children and as it’s worked out, my husband has taken on sort of more of the outside work and I’ve taken on more of the customer service and paperwork because I can do it with children.

This farmer’s experience illustrates how the arrival of children’s changing needs can upset even carefully-considered earlier plans, as well as how childcare needs can influence a farm’s labor structure.

##### Relationship and familial tensions

During interviews, gendered roles and family time emerged as critical issues related to childcare, most often mentioned by female participants. Female participants tended more often to express stress at their family’s gendered divisions of labor and discuss resulting issues in terms of their identities. Male participants tended to discuss the difficulties of work-family balance and the stress related to financial outlay of childcare. No men mentioned struggles of personal identity, deterioration of relationships, or feelings of isolation. However, as seven of the ten male participants were in focus groups, and one participated alongside his wife, group dynamics may have affected the gendered issues men felt comfortable speaking about. Nevertheless, differing perceptions of gendered labor divisions may foment or exacerbate tensions in farm households as it does in non-farm households (Frisco and Williams 2003).

Rural locations, self-employment, nuclear family patterns, and moving to access land can combine to create isolation. For one first-generation female participant who farmed as a sole proprietor, a missing social support network combined with insufficient financial resources to make childcare an isolating experience:

I’m completely by myself most days and it’s really hard to juggle. And so just the way our society is structured in a way. There isn’t that kind of strong support network of aunts and grandparents and sisters. And so…you have to supplement that with money. And farming just doesn’t bring in a lot. Farmers make low minimum wage a lot of times, but…then they have to pay above minimum wage [for childcare].

For this first-generation farmer–parent, her husband had grown up on a farm, but he had no interest in farming as an adult. While she admitted he would help when she “really need[s] it,” he had no love of agriculture and pursued his own creative passions through an off-farm career. They currently paid to keep their children in formal daycare or summer camps during the growing season, but she was considering putting the enrollment fees towards hiring farmworkers in future seasons. The cost of childcare frequently added stress to their relationship; she explained:

[My husband] sometimes thinks I should just quit farming, or get a job that’s going to actually pay more and pay for the childcare. I’m in this situation where the farm isn’t actually making an income yet, isn’t covering the costs. But it’s like I have to put in the time to make it get to the point where it can.

Even when couples do farm together, negotiating childcare and gendered household roles can add stress to relationships. After she took over more of the bookwork and indoor work of farming after the birth of their children, one woman lamented that her husband’s production skills had outpaced her own. “He just does everything so much faster than me now,” she said. “We’ve grown at a different pace.”

Other times, stress manifested when partners’ different experiences with care work led to divergent perspectives on how the household was functioning. During a focus group interview, a male farmer shared how his family successfully ran a diversified farming operation while home schooling three young children. His wife sat quietly while he proudly shared their accomplishments as a success story, but when he stepped out of the room, she opened up. From her perspective, the farm was near a breaking point. Nearly overwhelmed with the effort of homeschooling on top of agricultural production, she was unsure how much longer she could continue. The strain was palpable and left the moderators concerned about her mental health.

Left unchecked, this kind of tension can even lead to separation or divorce. One woman described how, when she farmed with her ex-husband, the layers of managing transportation needs, lacking childcare, and running a business had added up to too much stress for the marriage to bear.

Not having [childcare] makes it very difficult because we just can’t – we can’t do everything that we want to do. I have to run my business… as far as getting to the farm, it causes a lot of conflict. That’s probably why we’re not together anymore. It’s just like you can’t be dedicated to the business and the family and the partnership, all of that. It gets extremely overwhelming.

For some families, mental and physical health compounds the stress of organizing childcare. Paralleling international findings based on general population studies (Gray 2005; Low and Goh 2015), in our sample some farmers with aging parents discussed how they originally hoped their parents could care for their children, but in reality, their parents’ health limited their capacity to be helpful. One female farmer who relied on her mother for childcare described how, upon returning home from the fields, she found her daughter’s diaper had gone unchanged all day. She came to realize her mother suffered from dementia, yet she still needed her mother to watch her young daughter. She also shared her stress at her mother’s tendency to wander and become lost on the farm. The farmer had little knowledge of agencies, organizations or resources that could assist with the situation and found herself in the ‘sandwich generation,’ caught between taking care of her children, aging parents, and the farm operation.

For other families, a child’s disability limits options for appropriate care. One woman whose toddler had special needs could not find anyone in their community safely able to provide care. She and her husband carried the full load of childcare. Children with disabilities often require in-home care, and finding, much less affording, the necessary specialized care can be extra challenging for rural residents with disabilities (Iezzoni et al. 2006; Lishner et al. 1996). A child who requires extra care compounds the typical childcare conundrum and directly affects social relationships within the family, farm structure and management, and overall quality of life.

Even for participants deeply committed to raising their children on farms, accessing, arranging, and negotiating childcare introduces new social stresses that often fall along gendered lines. These stressors disproportionately affect female farmers, who continue to carry the bulk of physical and emotional care work on farms.

#### Economic

##### Farm organization and structure

On farms, childcare’s impacts extend beyond the relational into a range of economic realms. The relationship stresses, inequitable divisions of labor, and gendered care burdens discussed above affect farms’ day-to-day operations. Our interview data also show the ways in which childcare needs critically inform farmers’ decisions about structuring and running their operations. Childcare ripples outward from households to shape farmers’ business decisions, approach to organization, production, marketing, and growth trajectories. Such on-farm impacts vary according to individual families’ circumstances, values, and life-course position. Nonetheless, childcare consistently appears to underlie many physical and economic facets of farm operations.

Sometimes, farmers designed their operation’s physical organization intentionally with childcare in mind. One first-generation male farmer, who farms with his wife, described how they had planned their farm to have both a “near field” and a “back field.” Rather than reflecting their preferences around, for example, soil type or land slope, childcare needs motivated this plan. In the summers, their school-age son was old enough for his teenage sister to watch him in the house. But the parents still wanted to ensure the wife could easily check on them during the day, so she worked primarily in the “near field.” Another participant, transitioning her family’s farm to a growing schedule more conducive to spending time with her children in the summer, highlighted their significance, stating, “[They are] shaping the farm business in pretty significant ways – what I’m planning to grow and how to sell it.”

More generally, finding appropriate and affordable childcare affected some farmer–parents’ abilities to devote sufficient time to developing their farm business. One male participant described his unsatisfactory childcare situation and the difficulty of finding fulltime, summer childcare. As he saw it, dealing with and trying to remedy this situation was, “One of the things that’s kept us from going more full engine.” Childcare also directly influenced even the most basic business decisions about when one could work on the farm, with one female participant saying that her daughter’s needs “determine” her hours. Another participant went so far as to say, “Raising our kids… is included in my labor plan.”

Childcare can also influence labor decisions for farms that hire non-family workers. On farms with labor crews, remembering that their children would regularly interact with employees was a key part of the selection process. One participant stated that knowing her children would socialize with employees during the season made her, “Mindful of who we hire.” Another explained how she makes sure her crew members know that if her children want to join in, they are always welcome to, even if their involvement slows the work. For her, production efficiency could take a second place to encouraging her children’s interest in agriculture. Ensuring that her crew members were on board with this farm value was a crucial piece of managing labor.

For farmers with limited financial resources to hire help, deciding between hiring childcare or hiring farm labor often felt fraught. Hiring farm labor would let them spend time with their children on the farm, but childcare help would let them do the fieldwork themselves, more quickly and accurately than a typical employee. A male participant reported that he and his wife keep their children on their farm because of personal values, but they see non-parental childcare as a potentially more efficient use of financial resources:

… the cumulative effort of my wife and I minus a child would be a better worker than the money we’d be putting aside to pay that person with. It would be better to spend the money on childcare than to spend the money on an employee.

Although participants commonly recognize outside childcare as a financial investment that would improve productivity, agriculture’s low returns often made this choice unattractive. As one multi-generation female farmer explained the tradeoff, paying a babysitter the area’s going rate of $15 an hour to allow her to do fieldwork alone amounted to “paying to work.” “That’s hard to justify,” she concluded.

##### Childcare scheduling and transportation

As noted above, new farmers often move to land wherever they can afford it, which may mean more isolated areas requiring long commutes to childcare resources. For farmer–parents taking advantage of off-farm childcare, including camps, daycares, or schools, the strictness of pick-up and drop-off times frequently combined with this distance to negatively impact production and marketing.

One direct-marketer described how transporting her daughter to and from school interrupted her workday and “Took away from [her] ability to be as involved with the fieldwork as [she] wanted to be.” The stress of making daycare pick-up and drop-off times is surely one shared by non-farming parents, but the time-sensitivity of direct marketing opportunities is more particular to farmers. Non-profit organizations and other entities supporting agriculture often seek to cultivate new direct marketing opportunities for farmers, such as establishing new markets, food hubs, and farm-to-institution relationships. However, implementing these marketing channels without adequately considering family schedules can hinder their utility for farmer–parents. For example, one participant, who ran a diversified horticultural operation with her husband, spoke frankly about their decision to drop a market where they had registered as vendors. Although she could pick her daughter up from daycare in time to set up before the market’s 12 pm start time, the market manager refused to bend a rule requiring all vendors report on-site by 11 am. As the daycare only ran from 9 am to 11:30 am, picking up early enough to accommodate the manager’s rule made no sense for their logistics. They decided to withdraw as market vendors.

Another common scheduling conflict occurred because the growing season fundamentally conflicts with the school year. In the past, summertime school closures freed children to help their families with farm work; for many of today’s families, this schedule creates more conflicts. When parents do not expect or require labor from their children, and no family or household members can care for children, care arrangements must be made instead. Intense summer schedules and lighter winters can make finding appropriate care challenging, as expressed by one female farmer who farms with her husband:

… we don’t actually want to be sending [my son] off to daycare in January or February because I want to hang out with him and I have more time with him. And then in June and July when we totally need as much childcare as possible, everyone’s like no, we’re closed because everyone goes away… our schedule makes me feel like I work nights or something in relation to the universe.

##### Navigating the childcare assistance web

Federal and state-level subsidies aim to make formal childcare more affordable. Understanding how farm families draw upon this assistance helps present a more complete picture of the economic facets of childcare. Despite higher poverty and unemployment rates, rural families access childcare subsidies less frequently and for shorter periods of time than urban families (Davis et al. 2010). Our interview data suggest that some of this disconnect may be due to a mismatch between the qualifying criteria for subsidies and the realities of farming. One female farmer explained that her family had previously benefited from a state childcare subsidy. However, after her children’s father began working on the farm with her, they lost access to this support. As is the case on so many farms, the business could not afford to pay wages to family help. Without a wage and tax statement, her partner’s labor was rendered illegible to the bureaucratic system and, as the subsidy was earmarked exclusively for working parents, they lost access to support. Finding informal care for four children proved difficult, and the children now spent more time on the farm. This, she explained, reduced her farm’s productive capacity.

The participants who successfully accessed public subsidies to offset childcare costs unanimously described these as a boon to their farms, but other participants described frustration at finding out their finances situated them “just over the line” to qualify for benefits. Others described how the rigid and complicated system surrounding childcare subsidies effectively created a barrier between them and the support for which they qualified. One participant in Vermont admitted that she had received information on the state’s subsidy program but had not yet pursued it. “I should, but I haven’t gotten around to it,” she said. “I remember getting paperwork sent to me and then never filling it out. It’s just a tedious process. A lot of information.”

#### Cognitive

##### Waiting for the future to come

Childcare clearly impacts the social and economic facets of farm operations. Along a third dimension, we found that childcare arrangements also impacted how participants think about and plan their farm trajectories. *Cognitive* pathways linking childcare to farm operations appeared most frequently when farmers articulated the timeframes they use to reflect on their goals and farm structure. Many participants, envisioning farm growth and change, located these plans several years in the future, after their children grew out of the care-intensive early years. It seemed too hard to imagine growth, refinements, or changes in a farm’s operations in the near-term. Farmers, especially those with young children, spoke of the current time as a phase they needed to wait out before re-focusing once their children gained independence. For example, after describing how hard it was to get farm work done while also providing full-time care for his young sons, one man said, “And as they get older, they’ll be in public school. And so, I’m counting on that to help out, too, to give me some relief in the winter. And then in the summer, it’s nice enough outside and our farm is secure enough that they can just run around.”

Several participants described feeling as if, after initial years of investment, the need to now locate and coordinate childcare placed their anticipated farm growth on hold. One woman running an intensive vegetable operation with her husband frankly stated, “I would definitely say that childcare limits how much I intend to grow my business in the short term.” Sometimes this frustration occurred because farmers had found exciting opportunities for their farms to grow, but realized they had to wait several years before they could feasibly enact these plans. The immediacy of needing to care for young children kept them constrained to the homestead and reliant on the farm systems already in place. One participant, entering her sixth growing season, spoke about her plans to begin growing seedlings and perennials to diversify away from a primary focus on mixed vegetables. But when she considered how childcare played into her plans, she stated,

It’s limiting. Extremely limiting. At this point, I have great big visions and goals and my children are number one, so those have to be shelved, and it’s really sad because I have so many great – especially situated where we are. We have the potential to be a great community resource and now it’s a waiting game.

Other participants pointed towards imagined futures where their children might be able to assist with chores or be interested in working more intensively in agriculture, but felt like they had many years before they reached that potentially harmonious point. Noting that her school-age son had already started to express interest in the farm’s equipment, one female farmer commented,

I definitely believe that as we get older, there will be more chances, and like I said, we’re already beginning to see it where he can do the chores with my husband, where he will be a greater part of what we’re doing and it’ll be interesting to see how it evolves. I’m not quite sure how it’s going to evolve.

This woman expressed both hope that her son might maintain his interest in production and uncertainty at how that would play out.

In other times and places, farm families have laid plans guided by the assumption that their children would provide labor and take over productive activities once they were older. Without this overriding assumption, farmers can feel stymied. They must put their farm on hold during a child’s early years but cannot count on a labor return on this investment during the child’s teenage years. Children’s interest in farm work can change and evolve as they grow—an eight-year-old who relishes harvesting tomatoes with her mother does not guarantee a teenager who will help with deliveries. Childcare needs change over time, reflecting children’s age, interest, and abilities. Planning for these variables is also a part of farm business planning rarely discussed in whole farm planning guides.

Other participants with young children felt constrained in their ability to even imagine the contours of their future farms. Without knowing the extent to which their children would be interested in farm work, they struggled to know what groundwork to lay. When asked about how she hoped her school-age children would interact with the farm in the future, one female farmer hesitated, “I don’t know how to feel…It’s really hard to imagine what it’s going to be like once they’re a little bit older.” One farmer with a toddler son whose care was split between grandparents and a formal daycare center expressed cautious hopes that in a few more years, his son might be able to accompany him on farm chores: “I keep waiting for maybe when he gets older, I’ll keep him out of daycare all day on Tuesdays and Thursdays and he can just do deliveries with me.” Farmers spoke with guarded optimism that as their children gained independence, the work of integrating family and farm production would become easier. However, during a focus group interview, one woman with a daughter in Girl Scouts and other clubs cautioned that she now found herself off-farm more frequently than she had during the girl’s infanthood.

Although incompletely understood, these dynamics loomed large for the three participants without children. Their anticipated concerns and thoughtful planning revealed that childcare influences farm business planning even before pregnancy or childbirth. Cognizant of the wide, state-by-state variation in public support for working families, two participants actively debated the best states in which to search for farmland. As much as cost of land, soil quality, and familiarity with an area, public resources around health care and childcare weighed heavily in these future farmers’ decision-making. New and aspiring farmers are often fully aware that their business will likely be in the red for the first several years. This group openly shared that they consider public supports and subsidies key elements of attaining their twin goals of having a family and farming. One male participant in the early stages of establishing his farm explained, “We have no extra money in our budget now and we’re thinking of starting a business that’s going to be losing money. How would we find money for the childcare in the early years before they start school?” All three aspiring farmers had graduated from farmer apprenticeship programs during their early twenties. Now in their late twenties, their priorities were expanding to include the needs of spouses, partners, children and the ability to attain a comfortable standard of living.

The years during which farmers puzzle together childcare emerge as a distinct phase of contemporary farm development. Many farmers used temporal language when describing how they understood childcare to fit into their farm’s broader goals and future. One even went so far as to say, “I think the only thing that could make my situation more ideal is just the passage of time.”

## Discussion and conclusions

At a time of rapid, uncertain change in all areas of the U.S. agricultural sector, Braun’s (2019) attention to the nexus of extraordinary and ordinary on-farm stressors provides useful insights. Family farmers dealing with livelihood threats from global trade wars, severe weather events, and the COVID-19 pandemic are already experiencing severe stress. Household-level stressors amplify such challenges exponentially. Indeed, COVID-19 provides a timely illustration of the nexus of extraordinary and ordinary stressors: unpaid care work has increased drastically as a direct result of the pandemic, and emerging research indicates that, globally, women are unevenly shouldering this burden (Power 2020; United Nations 2020). The current uncertainty about school reopenings, relative risks and benefits, and children’s susceptibility to the coronavirus is further muddying childcare decision-making for farm and non-farm families alike. Efforts to support farmers are stronger when they recognize the full range of stressors to which farmers must respond. This article identifies and critically analyzes childcare as one type of ordinary stressor affecting farm families in the Northeastern United States.

We rooted our analysis in social science literature on the interplay between farm households and farm operations and on the gendered dynamics of farm households. Exploring the pathways that link childcare decisions and the farm operation reveals three pathways through which this household-level ordinary stressor impacts farms. Participants in this sample report that social relations on farms often change as partners negotiate childcare during a child’s young years. Several women described a decline in their own fieldwork activities, reporting they picked up additional book-keeping or administrative work conducive to staying close to young children. Familial and relationship tensions often followed. Childcare’s impacts extended beyond social worlds to impact a farm’s economics. Aside from the expense of childcare, participants reported decisions such as planning a farm’s infrastructure to accommodate childcare or dropping a market if they could not secure care for their children. Finally, needing to plan for childcare impacted the way farmer–parents conceptualized their operations’ trajectories. Like those interviewed by Dreby and Carr (2019), farmer–parents in this study clearly expressed their desire to raise children on farms. However, the joy of watching one’s children grow up on a farm sometimes exists at odds with logistical realities. The stress of negotiating childcare arrangements easily bleeds over from the farm household to the farm operation.

Government, non-profit organizations and private business have all made myriad investments to support agricultural producers. Recognizing and accommodating the gendered realities of parenting would help these investments better meet farmers’ needs. Currently, the majority of programming directed towards farmers focuses on goal setting, business plan development, financial management, record keeping, marketing practices, and low-cost, sustainable farming methods. These skills are critical during the early phases of the farm business enterprise. However, equally important yet rarely discussed in farm business planning curriculum are the social and life course events, such as having children, that affect the long-term viability and quality of life of the farm enterprise. Such issues are often at the root of farm business viability. Whole farm and holistic planning encourage farmers to situate their farm plans specifically in relation to personal and familial goals, recognize embodied skillsets, and account for potential risk factors to the farm operation that may lay outside the farm business. However, no comprehensive accounting of how childcare cost, availability or access affects farm structure, management or market strategies currently exists, nor how these dynamics may evolve as children age. Explicitly acknowledging issues such as childcare will better equip farmer–parents to handle the conflicting demands on their time and attention.

By naming children and care work as critical components of farm planning, these findings make basic household reproductive work more visible. As noted above, these activities have long been performed primarily by women and have long been systematically undervalued (Bianchi et al. 2012; Lachance-Grzela and Bouchard 2010). Households are dynamic entities whose members creatively cope with the policy and social landscapes on which they seek to make a living. Recognizing the fundamentally interconnected nature of farm households and operations empowers scholars and practitioners to better support resilient farmer livelihoods. Drawing on our research findings and parallel research examining the childcare needs of farmworkers, we see the need for programming that accounts for life course events (such as pregnancy) and childcare needs on farms. Training programs that incorporate a fuller array of pertinent household issues can provide farmers with the conceptual tools to better manage employees and more robustly envision and plan their own farms and families.

Historically, care work has remained largely invisible in both academic and practitioner approaches to farm economics and business planning. Farm households are not black boxes whose contents have unavoidable and unknowable impacts on farm operations. Farms have long been sites of both agricultural production and family reproduction. If scholars, practitioners, and policy makers continue to ignore the household and reproductive needs of farm families and farm workers, the United States risks accelerating farm exit rates at a time when the farm population stands at a precipice. Farming is an inherently risky and stressful occupation, and the negative impact of parental stress on children is widely documented (Bakoula et al. 2009; Williams Shanks and Robinson 2013). Farm children raised in high stress households may be more inclined to pursue non-farm careers as adults.

We recognize the specifics of childcare needs vary widely across social and geographic categories. As noted above, an important caveat to this article’s findings are that they only reflect the experiences of straight-passing, white farmers in the Northeast. White, heterosexual experiences have too often been taken as inherently generalizable. While we believe that the overarching pathways framework described in this article will prove useful for analyzing diverse farm household-farm operation relationships, the specifics will certainly vary. We strongly encourage future research to examine how childcare and other household issues differ for farmer–parents from different racial and ethnic backgrounds, of different gender and sexual identities, and of different citizenship statuses. These understandings will prove critical for supporting the entire U.S. farm population and building the next generation of farmers. Similarly, additional research is needed in regions where farm structure differs from the Northeast.

Future work should also investigate farmers’ and other rural residents’ openness to proposed policy and/or collective social changes that could help mitigate childcare-related stress. This study asked one open-ended question about what an “ideal childcare situation” would entail, but most participants could not articulate a concrete vision. Some mentioned living closer to family or the ability to hire a live-in nanny, but overwhelmingly, respondents seemed unable to imagine solutions to their childcare challenges. We recommend that researchers interested in undertaking this kind of research begin by outlining several possible interventions (e.g., public subsidies, models for nanny shares, universal guaranteed childcare) and then present them to participants for feedback. Future studies may do well to front-load the work of imagining change onto the research team, then use data collection to elicit responses and refinements from diverse farmer populations.

As food and agriculture scholars continue to integrate social issues into farm viability research, we must examine how broader national and state childcare policies intersect with farm family well-being and farm economic development. Expanding this vein through cross-national research would provide new insights into federal and sub-national program and policy priorities that can improve farm economics, farm family quality of life, and farmworker well-being. Pursuing these directions can help ensure researchers and practitioners do not advance one size fits all proposals. Such work is also relevant to broader rural economic development and rural wealth-creation initiatives. Efforts to understand childcare needs and effective strategies along the urban–rural continuum promise to yield findings relevant to many young families and address workforce attraction and retention across a range of industries.

## Footnotes

1 Interviews were scheduled if we could not arrange a minimum of three participants for a focus group. Ironically, farmers’ inability to participate was often related to childcare, despite the provision of on-site childcare.
